# Local Ordering, Distortion, and Redox Activity in (La_0.75_Sr_0.25_)(Mn_0.25_Fe_0.25_Co_0.25_Al_0.25_)O_3_ Investigated by a Computational Workflow for Compositionally Complex Perovskite Oxides

**DOI:** 10.1021/acs.chemmater.3c03038

**Published:** 2024-05-13

**Authors:** Boyuan Xu, Jiyun Park, Dawei Zhang, Héctor A De Santiago, Wei Li, Xingbo Liu, Jian Luo, Stephan Lany, Yue Qi

**Affiliations:** † Department of Physics, Brown University, Providence, Rhode Island 02912, United States; ‡ School of Engineering, Brown University, Providence, Rhode Island 02912, United States; § Program in Materials Science and Engineering, University of California San Diego, La Jolla, California 92093, United States; ∥ Department of Mechanical, Materials and Aerospace Engineering, Benjamin M. Statler College of Engineering and Mineral Resources, West Virginia University, Morgantown, West Virginia 26506, United States; ⊥ Department of NanoEngineering, University of California San Diego, La Jolla, California 92093, United States; # Materials Science Center, National Renewable Energy Laboratory, Golden, Colorado 80401, United States

## Abstract

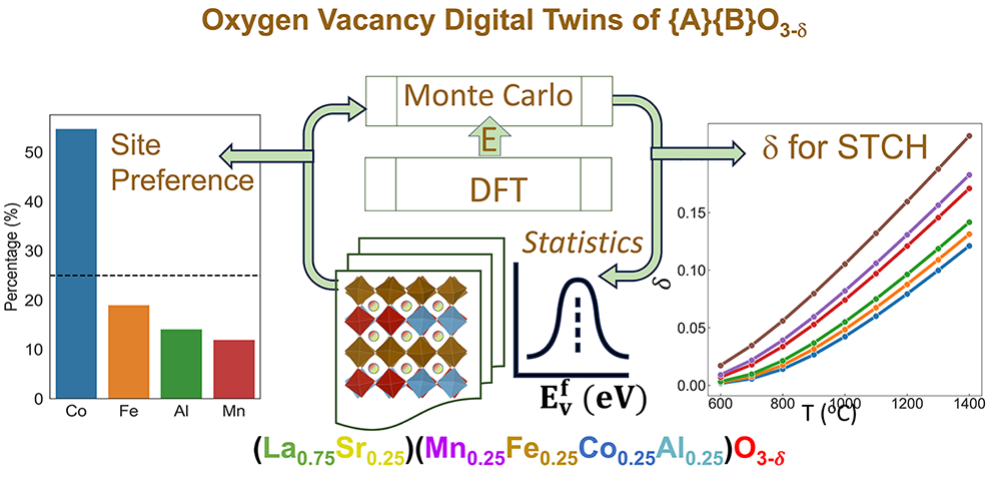

Mixing multiple cations can result in a significant configurational entropy, offer a new compositional space with vast tunability, and introduce new computational challenges. For applications such as the two-step solar thermochemical hydrogen (STCH) generation techniques, we demonstrate that using density functional theory (DFT) combined with Metropolis Monte Carlo method (DFT-MC) can efficiently sample the possible cation configurations in compositionally complex perovskite oxide (CCPO) materials, with (La_0.75_Sr_0.25_)(Mn_0.25_Fe_0.25_Co_0.25_Al_0.25_)O_3_ as an example. In the presence of oxygen vacancies (*V*_O_), DFT-MC simulations reveal a significant increase of the local site preference of the cations (short-range ordering), compared to a more random mixing without *V*_O_. Co is found to be the redox-active element and the *V*_O_ is the preferentially generated next to Co due to the stretched Co–O bonds. A clear definition of the vacancy formation energy (*E*_v_^f^) is proposed for CCPO in an ensemble of structures evolved in parallel from independent DFT-MC paths. By combining the distribution of *E*_v_^f^ with *V*_O_ interactions into a statistical model, the oxygen nonstoichiometry (δ), under the STCH thermal reduction and oxidation conditions, is predicted and compared with the experiments. Similar to the experiments, the predicted δ can be used to extract the enthalpy and entropy of reduction using the van’t Hoff method, providing direct comparisons with the experimental results. This procedure provides a full predictive workflow for using DFT-MC to obtain possible local ordering or fully random structures, understand the redox activity of each element, and predict the thermodynamic properties of CCPOs, for computational screening and design of these CCPO materials at STCH conditions.

## 1 Introduction

“Solar-to-fuel” techniques take advantage of the whole solar spectrum to produce sustainable energy sources, such as natural gas,^1,2^ hydrogen and syngas,^3,4^ and biofuels.^5−7^ Two-step solar thermochemical hydrogen (STCH) generation is one of the proposed technologies to generate hydrogen by splitting water with an essential material through reduction and oxidation reaction cycles. Due to their tolerance of high oxygen nonstoichiometry, perovskite oxides (ABO_3_) have been pursued for the STCH process, which can be written in two thermochemical redox reactions as^8,9^
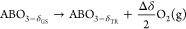
1
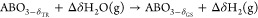
2where δ_TR_ and δ_GS_ are the oxygen nonstoichiometry at the thermal reduction (TR, high temperature) and gas-splitting/oxidation (GS, low temperature) conditions, respectively. Δδ = δ_TR_ – δ_GS_ gives the oxygen exchange capacity, thus is a thermodynamic parameter that determines the hydrogen production occurrence and capability.^9−11^ ABO_3_, with a broad range of oxygen vacancy formation tunability by accommodating different types of cations on A- or B- sites, fulfills its application in various fields, including STCH generation.^12,13^ More than 5000 single-component perovskite compounds^14^ have been computationally screened to explore STCH potential candidates with oxygen vacancy formation energy close to the benchmark material, nonstoichiometric CeO_2_, which had successfully demonstrated more than 500 STCH cycles.^15−17^

Cation configurational entropy-stabilized single-phase compositionally complex perovskite oxides (CCPOs) with four or more cations mixed on the A and/or B sites can tolerate a larger fraction of oxygen vacancies while maintaining the perovskite structure in comparison with their single-component counterparts. Several CCPO combinations were reported to have extraordinary performance in water-splitting-related applications, such as (Pr_0.2_Ba_0.2_Sr_0.2_La_0.2_Ca_0.2_)CoO_3−δ_,^18^ (La_0.6_Sr_0.4_)(Co_0.2_Fe_0.2_Mn_0.2_Ni_0.2_Mg_0.2_)O_3_,^19^ and La(Cr_0.2_Mn_0.2_Fe_0.2_Co_0.2_ Ni_0.2_)O_3−δ_.^20^ One of the good performing STCH composition of (La_0.8_Sr_0.2_)(Mn_(1–*x*)/3_Fe_(1–*x*)/3_Co_*x*_Al_(1–*x*)/3_)O_3_ (denoted as LS_MFC_*x*_A) demonstrated ∼400 μmol/g within 1 h cycling time, as reported by Zhang et al. in a recent paper.^21^ In addition to promoting thermodynamic stability by cation mixing-induced configurational entropy, the redox activity is also important. It was demonstrated that not all of the B-site elements in LS_MFC_*x*_A are redox-active. The combined computational and experimental work revealed that Co is a redox-active element. The Co site preference for oxygen vacancy formation in LS_MFC_*x*_A can be attributed to the stretched Co–O bonds. Thus, redox activities of different elements and their local chemical and bonding environments must be considered when designing and optimizing the CCPO composition for STCH generation.

The introduction of multiple cations not only offers a new compositional space with vast tunability but also introduces new computational challenges due to sampling. Most first-principles studies assume a highly disordered or fully random distribution of multiple cations in CCPOs that can maximize configurational entropy. Special quasi-random structures (SQS) proposed by Zunger et al. is a commonly used method to build a random solid solution density functional theory (DFT) model to maximize the randomness in supercells with limited sizes.^22,23^ It has been applied to multiple compositionally complex systems of carbonates,^24,25^ borides,^26^ and perovskites^27^ in calculations. The SQS method aims to optimize the atomic random distribution with a minimum supercell by minimizing the correlation functions, where the chemical species occupation is treated as equal. For the A-site mixing case in CCPOs, the random assumption is likely to be true since the oxygen vacancy formation energy (*E*_v_^f^) is not very sensitive to the elemental species on the A-site but is only sensitive to the A-site oxidation state. However, *E*_v_^f^ is very sensitive to both B-site elemental species and oxidation states, and the *E*_v_^f^ difference could vary by more than 5 eV for different B-site species with the same charge on the A-site.^28^ Therefore, it is likely that the elemental distribution around an oxygen vacancy (*V*_O_) might not be random, but rather show some preference, which is called “local-order” around oxygen vacancies.

In order to answer the questions with respect to vacancy formation and determine the representative structures with or without possible local ordering in CCPOs, we implemented a density functional theory (DFT)-based Metropolis lattice Monte Carlo (DFT-MC) sampling workflow, inspired by a previous DFT-based crystal structure search method,^29^ to investigate the system with and without *V*_O_ at a given temperature. Our previous joint modeling and experimental paper^21^ only reported DFT-MC sampling results starting from an experimentally observed LS_MFC_*x*_A space group, which is trigonal (*R*3̅*c* space group 167) and it can be found in LaAlO_3_^30^ and LaCoO_3_.^31^ However, perovskite oxides, especially with cation mixing, exhibit a variety of symmetries. The most common form is cubic (*Pm*3̅*m*, space group 221), such as SrFeO_3_.^32^ When the A and B size ratio changes, the cubic perovskite structure distorts by tilting the BO_6_ octahedra and displaces the A-site atoms. The distorted structures include orthorhombic (*Pnma*, space group 62) such as LaFeO_3_^33^ and LaMnO_3_,^34^ and hexagonal (*P*6_3_/*mmc* space group 194) SrMnO_3_ structures.^35^ It becomes complicated to mix these structures into a CCPO. The first question that needs to be addressed is the structure of the CCPO. For the randomly A-site mixed {A}FeO_3_ CCPO, Park et al. showed that as the number of A-site species increases, especially above four types, the cell becomes more cubic-like while the local Fe–O octahedra are more distorted.^36^ In this paper, we present a more general and systematic DFT-MC sampling workflow, starting from multiple low-energy ABO_3_ structures of different symmetries with the goal of determining the lattice symmetry of CCPO.

The DFT-MC sampling method leads to a group of sampled structures at a given composition, and questions arise when defining the oxygen vacancy formation energy (*E*_v_^f^) of this CCPO. A clear definition is critical since *E*_v_^f^ has been utilized as a screening criterion in many DFT-based phenomenological models to identify new candidate materials for applications including STCH.^28,37,38^ Typically, *E*_v_^f^ is defined as the energy difference between the vacancy structure and its pristine bulk structure. In cation-mixed CCPO, it is inevitable that the oxygen vacancy formation energy exhibits a statistical distribution. Given a parent structure, the *E*_v_^f^ on different oxygen sites can be sampled to give a distribution, as in many simulation studies.^36,39^ However, the DFT-MC method will sample the cation configurations around a vacancy; thus, there is no parent structure to define the energy difference. We propose to obtain the oxygen vacancy formation energy distribution based on the sampled bulk and vacancy structure energy distributions. Furthermore, we incorporate the *E*_v,CCPO_^f^ with a DFT-informed statistical model for accurate prediction of oxygen vacancy nonstoichiometry, δ, under STCH conditions proposed by Park et al.^36^ The model connects the DFT-calculated oxygen vacancy energy distribution with the oxygen chemical potential (μ_O_) at the operating temperatures and oxygen partial pressures to estimate δ at STCH conditions and could be compared with the experimental thermal gravimetric analysis results. Building on the partial success of DFT-MC simulations on LS_MFCA, this work presents a complete workflow to predict Δδ for CCPO in STCH applications.

This paper is organized as follows: First, a workflow to predict oxygen vacancy nonstoichiometry, δ, in compositionally complex oxides from the DFT-MC sampling method and the statistical model is presented. We focus on LS_MFCA as an example. For simplicity, a 3:1 ratio at the {A} site, and an equimolar ratio at the {B} site, i.e., (La_0.75_Sr_0.25_) (Mn_0.25_Fe_0.25_Co_0.25_Al_0.25_)O_3_ (denoted as LS_MFCA), are selected for DFT calculations. The randomness and local ordering in the bulk and oxygen vacancy-containing structures are analyzed. The role of each cation species in terms of their local oxidation states and bond distortions is examined to shed light on the compositional design of the CCPO. Finally, the enthalpy and entropy of reduction are extracted from the predicted δ using the van’t Hoff method for direct comparison with experiments.

## 2 Computational Methods

**Figure 1:**
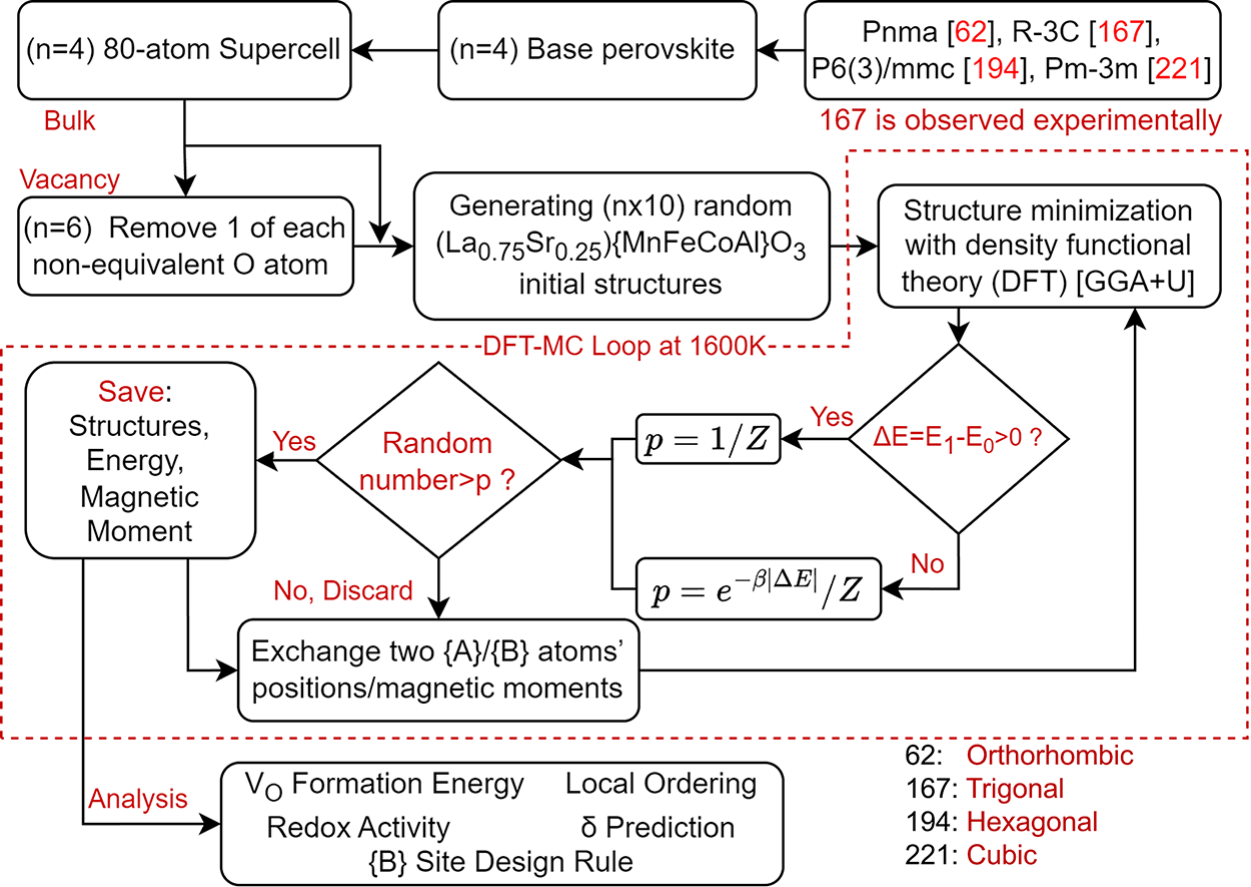
DFT-MC workflow to efficiently sample the possible cation configurations and discover the role of each {B} site element in oxygen vacancy formation in the LS_MFCA material as an example. Bulk and vacancy structures follow parallel sampling paths to ensure their independence. Typical perovskite symmetries, *Pnma* (SG 62), *R*3̅*c* (SG 167), *P*63/*mmc* (SG 194) and *Pm*3̅*m* (SG 221), marked by their space group (SG) numbers, are selected as base structures.

Figure 1 shows the workflow used to sample the large structural space of CCPOs. We performed DFT-MC simulations for both the bulk and vacancy-containing LS_MFCA structures. The four most common perovskite symmetries with representative compounds, LaMnO_3_*Pnma* (SG 62), LaAlO_3_*R*3̅*c* (SG 167), SrMnO_3_*P*6_3_/*mmc* (SG 194), and LaMnO_3_*Pm*3̅*m* (SG 221), were used as the initial perovskite structures (Figure S1a–d shows the representative initial structures) to generate 80-atom supercells (Figure S1e–h). These structures were taken from the NREL materials database, which have been benchmarked with GW electronic structure calculations.^40,41^ As a balance between accuracy and sampling efficiency, the size of the supercell (containing 80 atoms as La_12_Sr_4_Mn_4_Fe_4_Co_4_Al_4_O_48_, cell length: ∼11 Å, vacancy concentration: 2.1%) is chosen so that the distance between the oxygen vacancy and its image is larger than 10 Å to reduce the vacancy interaction, and the defect formation energy has been shown to be converged within ∼0.1 eV in simple ABO_3_ cases.^42,43^ Ten random “seed” structures (cation-randomly mixed LS_MFCA supercells, examples for each symmetry are shown in Figure S1i–k) were generated by homemade codes to randomize both A and B cations assuming that they are fully random on their sublattices for each symmetry according to the elemental ratio. A test run with both atomic and volume relaxation is conducted first over about 50 MC iterations; within each iteration, the simulation cell, ionic, and electronic structures are fully relaxed. The average volume is then determined for the subsequent DFT-MC sampling, which, at that point, includes only atomic relaxation to speed up the computations. We tested and confirmed that the equilibrated MC structures were volume-relaxed, obtaining a mean absolute pressure of only 4.3 kbar over 6 samples. Each seed structure evolved following an MC “path” by alternating atomic and magnetic swaps. Atomic swaps choose a random cation pair but only allow swaps that maintain the correct A (La, Sr) and B (Mn, Fe, Co, Al) site occupation. Magnetic sampling swaps a spin-up/down pair on the B-site sublattice, thereby maintaining an average paramagnetic configuration. The swaps are followed by DFT calculations with atomic relaxation, and the Metropolis MC acceptance criterion at 1600 K with the DFT-computed energies. This specific setup aims to sample the space of atomically and magnetically disordered LS_MFCA structures at typical experimental thermal reduction temperatures of around 1350 °C. For the vacancy-containing structures, one vacancy was created by removing one of the nonequivalent oxygen atoms from the supercell of the representative initial seed structures. These defect structures evolved following an analogous MC “path” for configuration sampling. Thus, importantly, cation configurations are sampled independently in structures with and without vacancies. This approach is different from forming defects after determining the cation disorder (e.g., by SQS construction), in that it includes short-range order effects induced by *V*_O_ defects. The accepted bulk and vacancy-containing structures were used for the analysis.

Plane wave-DFT calculations were performed using the Vienna Ab Initio Simulation Package (VASP).^44^ Projector augmented wave (PAW) potentials and the valence electrons configuration for each element are La (5s^2^5p^6^6s^2^5d^1^ of the valence electronic configuration), Sr_sv (4s^2^4p^6^5s^2^), Mn (3d^5^4s^2^), Fe (3d^6^4s^2^), Co (3d^7^4s^2^), Al (3s^2^3p^1^), and O_s (2s^2^2p^4^). To account for multiple valences and spin states at the STCH operating temperatures (1100–1600 K in experiments above the Néel temperature), even though some materials have ferromagnetic ground states at low temperatures, the input magnetic moments are selected randomly to be half-up and half-down for the {B} site elements to be paramagnetic.^45^ The generalized gradient approximation (GGA) of Perdew, Burke, and Ernzerhof (PBE) was used for the DFT-exchange correlation functional.^46^ The Hubbard *U* correction for 3d transition metal (Co, Mn, Fe) electrons was chosen to be 3.0 eV,^47^ and *U* values for La 5d and 4f electrons were set to be 1.5 and 2.0 eV, analogous to the parameters previously determined for Ce.^29^ The plane-wave cutoff energy was set to be 320 eV, which was shown to be sufficient for the soft oxygen pseudopotential by Peng et al.,^48^ who used 320 and 500 eV energy cutoff for soft and standard version PAW potentials of O, respectively, to explicitly compare defect formation energies and elemental energies that leads to less than 0.02 eV differences. A 2 × 2 × 2 γ point Monkhorst–Pack *k*-point mesh was used for all calculations. The electronic and atomic relaxation convergence criteria were 8 × 10^–5^ and 0.04 eV Å^–1^, respectively. These settings provide a reasonable compromise between accuracy and efficiency for a large number of MC trial calculations. A comparison between this convergence setting and a more accurate setup (1 × 10^–6^ and 0.01 eV Å^–1^ with cutoff energy set to 350 eV) was conducted for two randomly selected structures from each of SG 62, SG 167, and SG 221 symmetry, and the energy differences between these two criteria are within 0.06 eV for all selected structures. The other DFT + *U* setup was tested (details in Section S2), and the qualitative trend in *E*_v_^f^ with vacancy concentration was shown to be independent of *U* selection.

The DFT-MC sampling generates energy distribution for both bulk and vacancy-containing structures and are fitted with a Gaussian function to obtain average *E̅* and standard deviation σ. The *E*_v_^f^ distribution for CCPOs takes a larger deviation from the two distributions and is defined as
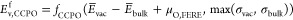
3where *f*_CCPO_ is the estimated *E*_v_^f^ distribution for the CCPO in the Gaussian form with the mean and standard deviation calculated in parentheses. μ_O,FERE_ is the fitted elemental reference energy for oxygen.^47^

Using the distribution *E*_v,CCPO_^f^, the oxygen deficiency δ can be predicted as described in the Method Section of Park et al.^36^ High oxygen vacancy concentrations at the TR conditions can introduce oxygen vacancy interactions, such that the oxygen loss is accompanied by an increase in *E*_v_^f^, as found previously in SrFe_0.75_Mo_0.25_O_3_ and SrFeO_3_. Thus, an oxygen vacancy interaction term should be considered (see below).^49,50^ Each oxygen site in a structure together with its local cations can be considered a subsystem, and *E*_v,i_^f^ is the energy required to form a *V*_O_ at the *i*th oxygen site. If *V*_O_ occurrence at each available oxygen site is considered an independent event, the *V*_O_ interaction can be assumed to take a linear form and does not affect the distribution shape. The *V*_O_ formation probability for such a site can still accommodate the equation for a two-level system. To obtain the total O deficiency δ, we used a discretized distribution function *f*_CCPO_(*E*_v,*i*_^f^) for the relative fractions of O sites with a corresponding formation energy *E*_v,*i*_^f^.
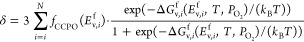
4where *k*_B_ is the Boltzmann constant and *N* is the number of discrete energies used for the summation (in this paper, *N* = 2000 is used). The free energy of *V*_O_ formation at a given temperature *T* and pressure *P*_O_2__ can be connected with the *E*_v_^f^ formation energy by a modified oxygen chemical potential term Δμ_O_(*T*,*P*_O_2__), describing the chemical potential change of the O_2_-gas phase from 0 K to a given temperature *T* at *P*_O_2__:^50,51^
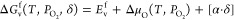
5Further, Δ*G*_v_^f^ includes the linear term α·δ, describing the concentration-dependent repulsive vacancy interactions due to local bond distortions and excess electrons associated with *V*_O_ formation.^50,52^ To determine the vacancy interaction coefficient α for the Co^3+^/Co^2+^ redox pair, we used a series of separate calculations for LaCoO_3_ supercells of varying sizes, from 40, 80, and 160 atoms (see details in Section S2). As shown in Figure S3a, the *E*_v_^f^ increase in this range is close to linear. The linear fitting of the DFT-MC setup (marked in blue) gave an interaction coefficient of α = 5.66, with the overall trend of *E*_v_^f^ increasing quickly at δ < 0.125 and then flattening out after this value.

## 3 Results

### 3.1 Energy Evolution, Vacancy Formation Energy, and **δ** Prediction

**Figure 2:**
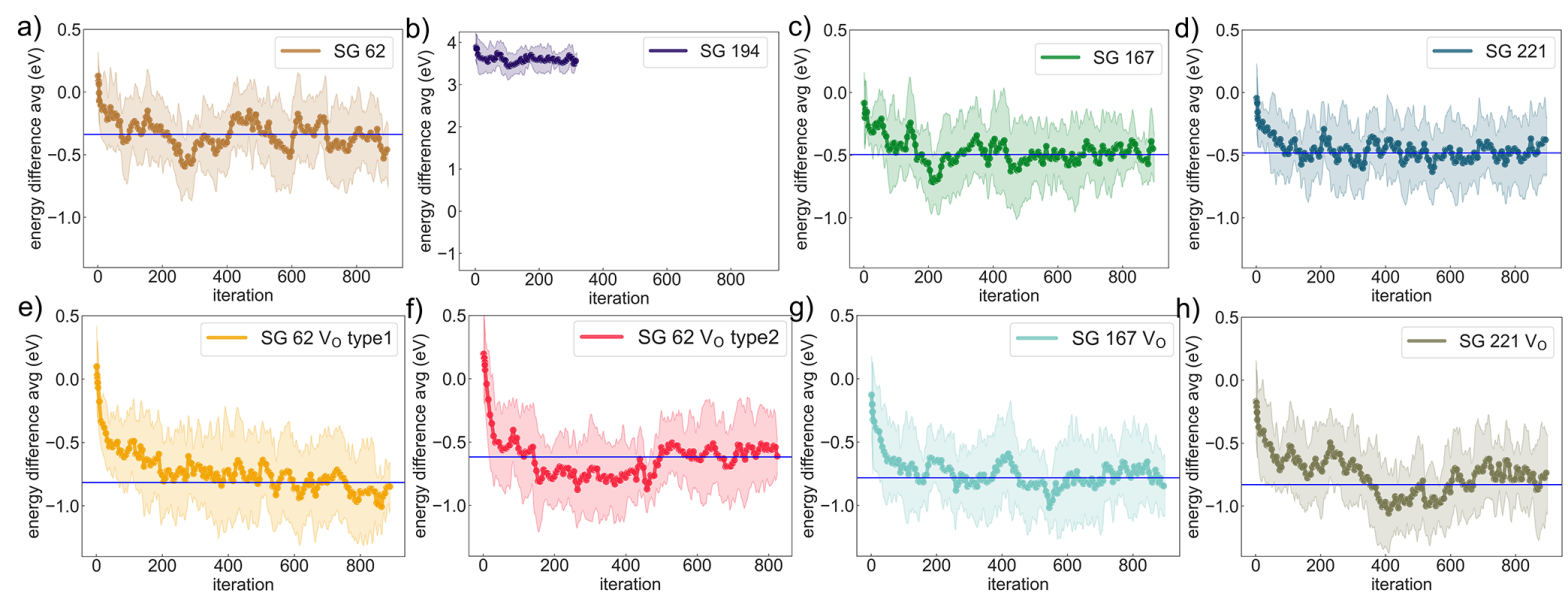
Energy evolution plot. Average of every 5 energy points along the MC path over all 10 seeds with MC iterations for (a–d) bulk and (e–h) vacancy structures. Nonequivalent O sites are considered for SG 62 and are labeled as *V*_O_ type1 and *V*_O_ type2 in (e, f). SG 194, the hexagonal seed has around 0.22 eV per formula unit higher energy than the other symmetries and thus is omitted in the discussion. For other symmetries (SG 62, 167, 221), the stable stage is reached after 400 iterations, and their average energies after the stable stage are marked as horizontal solid blue lines.

The energy evolution behavior provides an overview of the DFT-MC proceeding status. Initially, 10 seeds were used for each symmetry. The reference energy −594.14 eV (−586.97 eV) of the simulation cell for bulk (vacancy containing) configurations is calculated by averaging the energies of all 10 seeds for SG 62, SG 167, and SG 221 initial random bulk structures (40 for SG 62, SG 167, and SG 221 derivative vacancy structures). It is taken as the 0 eV point on the *y*-axis in Figure 2, and all energies reported are relative energies with respect to this point. The SG 194 structures, with mixed face and corner-sharing octahedrons, were excluded from the average due to their much higher energies than the other three symmetries with only corner-sharing octahedrons.

As the structures evolved following their DFT-MC paths, the energy evolution was reported for each symmetry by averaging every 5 energy points along the MC path over all 10 seeds. Only the energy of accepted structures will be updated; otherwise, the energy of that iteration is kept the same as in the last step. The energy evolution with respect to iterations for each symmetry is plotted in Figure 2, where the shaded area shows the standard deviation processed in the same way. As the MC simulations proceeded, the energy decreased quickly and then fluctuated after a maximum of 400 iterations. An overall energy gain of 0.3–0.5 eV for the bulk sampling path and 0.6–0.8 eV energy gain for the vacancy sampling path were observed. The “stable” stage was reached when the average deviation of the energy in each seed along the MC path did not result in a decrease in averaged energy but led to a fluctuation behavior. The converged energies, marked by solid blue lines, were obtained by averaging all stable-stage energies for each symmetry. Figure 2b shows that SG 194, the hexagonal symmetry, has around 0.22 eV per formula unit higher energy than the rest of the symmetries and thus is aborted after 300 iterations and omitted in the following discussion with no converged energy calculated. Since the stable-stage structures from SG 62, SG 167, and SG 221 do not show a distinguishable difference in energy, we chose to report our calculations, findings, and statistics for these structures as a whole in the following discussions. SG 194 was no longer considered.

**Figure 3:**
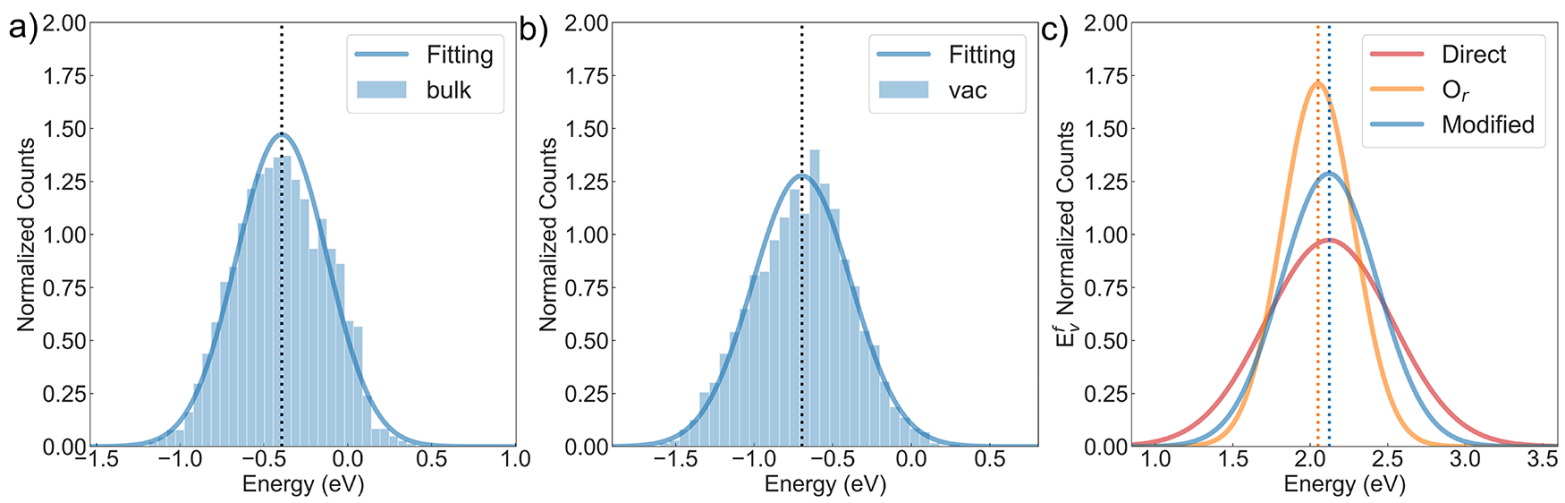
Energy distribution of accepted stable-stage (a) bulk and (b) vacancy structures. The fitted Gaussian functions have energy averages at −594.54 and −587.66 eV with standard deviations of 0.27 and 0.31 eV, respectively; (c) vacancy formation energies obtained by the direct (red), O refilled (O_r_, orange) and modified (blue) methods. They are fitted by Gaussian functions, which are shown in the same figure. The distribution centers lie at 2.12, 2.05, and 2.12 eV with standard deviations of 0.41, 0.24, and 0.31 eV, respectively.

The accepted structures saved after 400 iterations were considered to reach a stable stage, and a total of 2600 bulk and 3000 vacancy saved structures were treated as possible LS_MFCA configurations and selected for further analysis. The energy distribution of stable-stage accepted bulk (vacancy) structures with respect to the reference energy for the simulation cell is plotted in Figure 3a (Figure 3b) and a Gaussian function is fitted to it, yielding an average and standard deviation of −594.54 ± 0.27 eV for the bulk structure and −587.66 ± 0.31 eV for the vacancy-containing structure.

The traditional method for calculating the oxygen vacancy formation energy *E*_v_^f^ requires the energy difference between a vacancy-containing structure and its pristine bulk structure. However, the independent and parallel evolution of the bulk and vacancy structures through DFT-MC cannot fulfill these requirements. Multiple solutions to define *E*_v_^f^ for LS_MFCA are possible. A simple approach is to directly take the distribution subtraction: the average follows *E̅*_v,CCPO_^f^ = *E̅*_vac_ – *E̅*_bulk_ + μ_O,FERE_, the standard deviation comes from  (Figure 3c, red line, named the direct method). This method tends to propagate/broaden the distribution. Alternatively, we can refill the vacancy structures by putting the oxygen atom back (energy distribution in Figure S2) and calculating the refilled vacancy formation energy *E*_v,r_^f^, as shown by the orange line in Figure 3c. Such a refilling operation provides a well-defined defect-host energy difference, but the newly created host structures may not be a thermodynamically valid representation of the defect-free oxide if the *V*_O_ defect introduces preferential cation coordination, which is the case in LS_MFCA, as discussed below. In this case, *E*_v,r_^f^ underestimates the vacancy formation energy. Third, we proposed a simpler method in eq 3, which led to another *E*_v_^f^ distribution for CCPOs (Figure 3c, blue line, named the modified method). The differences for all three definitions, direct, O refilled (O_r_), and modified methods, are compared in Figure 3c and are shown in red, orange, and blue, respectively. It can be seen that (1) the refilled *E*_v,r_^f^ distribution gives the narrowest Gaussian peak that sits at 2.05 eV with a standard deviation σ_r_ = 0.24 eV, lower than *E*_v,d_^f^ as we anticipated; (2) the direct distribution estimates an average *E*_v,d_^f^ of 2.12 eV and a wider σ_direct_ = 0.41 eV; and (3) our proposed modified distribution estimation results in the same 2.12 eV average *E*_v_^f^ as a direct method but with an intermediate σ_modified_ = 0.31 eV. The error propagation during subtraction introduces a 70% larger standard deviation when compared to the oxygen-refilled case. A larger distribution width will lead to an overestimation of the oxygen vacancy nonstoichiometry.^36^ Thus, the modified method in eq 3 would be an improved method, as both its average and deviation are less than the 0.07 eV difference compared to the *E*_v,r_^f^ distribution.

**Figure 4:**
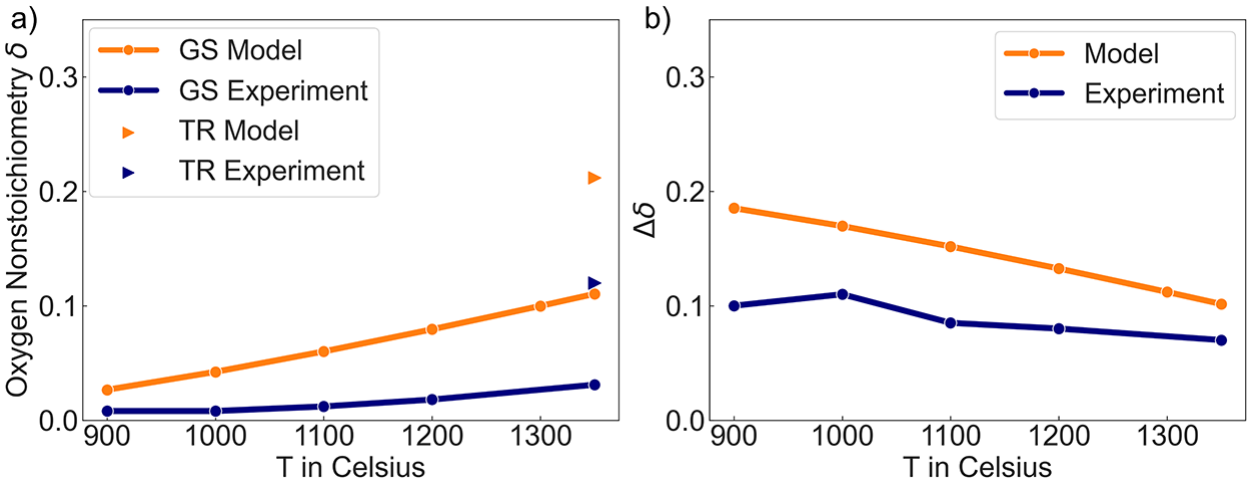
Comparison of the statistical model: predicted and experimentally measured *V*_O_ nonstoichiometry δ_TR_ and δ_GS_. Experimental dry TGA measurements of the LS_MFCA material with a Co content of 0.25 are marked in dark blue. (a) The TR condition is 1350 °C with *P*_O_2__ = 10^–5^ atm and marked as triangles, while the dry GS condition has a varied temperature range from 900 to 1350 °C with *P*_O_2__ = 0.21 atm and is marked as line and dots. (b) The experimental Δδ trend is directly compared with the model-predicted Δδ trend.

The resulting *V*_O_ formation energy distribution *E*_v,CCPO_^f^ was used as input for the high-entropy interacting (HE-Int) model to predict the oxygen vacancy nonstoichiometry (δ) following Park et al.^36^ This model has been used to predict δ and Δδ for Nd_0.5_La_0.5_FeO_3_, Sr_0.5_La_0.5_FeO_3_, Ba_0.5_La_0.5_FeO_3_, and Nd_0.25_Sr_0.25_Ba_0.25_La_0.25_FeO_3_,^36^ and showed the best agreement with experiments compared to other models that do not consider the oxygen vacancy formation energy distribution and oxygen vacancy interactions simultaneously. The model predicts δ at different conditions, as shown in Figure 4. Specifically, the TR condition is 1350 °C with *P*_O_2__ = 10^–5^ atm and the dry GS condition has a varied temperature range from 900 to 1350 °C with *P*_O_2__ = 0.21 atm. δ_TR_ and δ_GS_ are compared with the experimental dry TGA measurements of Co content of 0.25,^21^ as shown in Figure 4, The predicted Δδ values have a similar temperature dependence as the experimental data, but the δ values systematically overestimate O deficiency, e.g., by a factor 2 under TR conditions.

The reasons for this discrepancy are convoluted and may originate from the DFT accuracy and incomplete theoretical models. Figure S3a shows 0.2 eV higher *E*_v_^f^ for LaCoO_3_ by using another DFT setting (setup1, different U selections with standard oxygen pseudopotential and higher cutoff). If we assume that such behavior is preserved for LS_MFCA *E*_v_^f^, it would have resulted in an even lower δ prediction, as shown in Figure S3b; however, this does not change the Δδ results (Figure S3c), indicating that the discrepancy is not solved. The vibration entropy contribution is less than 0.1 eV on *E*_v_^f^ based on similar systems; thus it cannot be responsible either.^50^ We ascribe the other part of the discrepancy to the simplified assumption of independent oxygen vacancy occurrence at each site, as pointed out in the original paper. With the increase of *V*_O_ in the system, redox-active sites are depleted, thus raising the *E*_v_^f^ and changing its distribution shape simultaneously. The former impact is included in the model and simplified as a linear term α·δ, but the latter effect is not incorporated in the current model. We would leave the improvement of the model to further studies.

### 3.2 Random Bulk Structures and Locally Ordered Vacancy Structures

**Figure 5:**
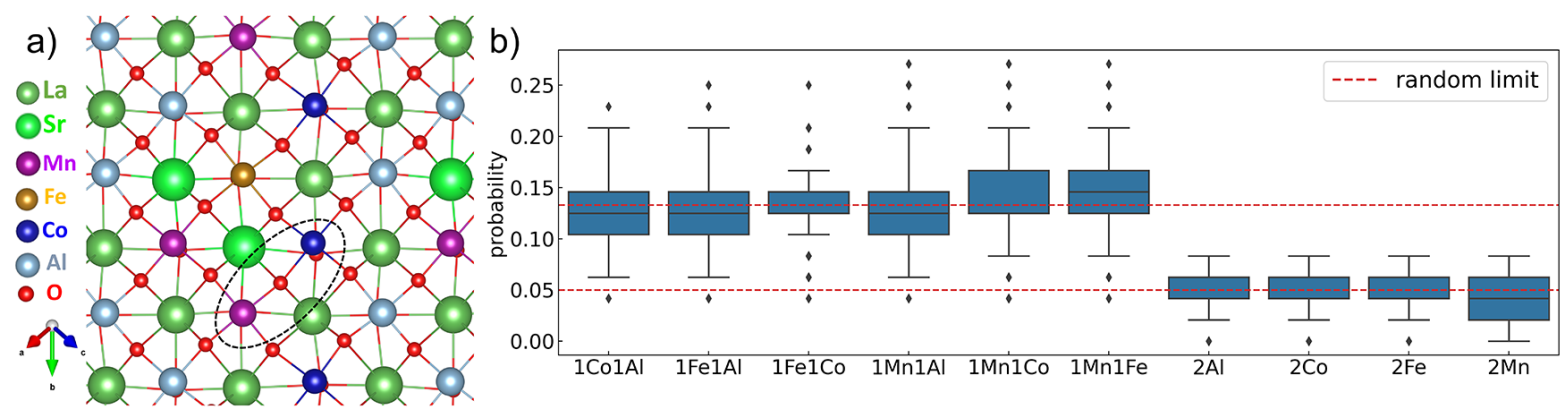
(a) Local ordering is defined as O FNN {B} site element combinations, one example of Mn–Co in the [101] view is circled. (b) O FNN {B} combination statistics for accepted stable-stage bulk structures; red dotted lines represent random limits.

We next consider the possible local ordering that may be unveiled by our DFT-MC method, and some of the results for symmetry 167 have already been reported in ref; (21) here, we reported the averaged data for the symmetries of SG 62, 167, and 221 since they have similar energies. In a perovskite structure, the oxygen atom is surrounded by four first nearest-neighbor (FNN) {A} site atoms and two FNN {B} site atoms. Here, the local ordering is defined as oxygen FNN {B} site element combination, and this combination is denoted as two {B} site elements connected by a hyphen symbol. An example of Mn–Co is circled in Figure 5a in the [101] view. For totally random structures in an LS_MFCA 80-atom cell, the probability of finding O FNN atoms of the same type of B element is , and that of finding different B elements B_1_–B_2_ is . These two random limits are plotted in Figure 5b as dotted red lines. The combination distributions for the accepted bulk structures shown are around the random limit with no preferred terms. Thus, we claim that no special preference is found in the bulk configurations.

**Figure 6:**
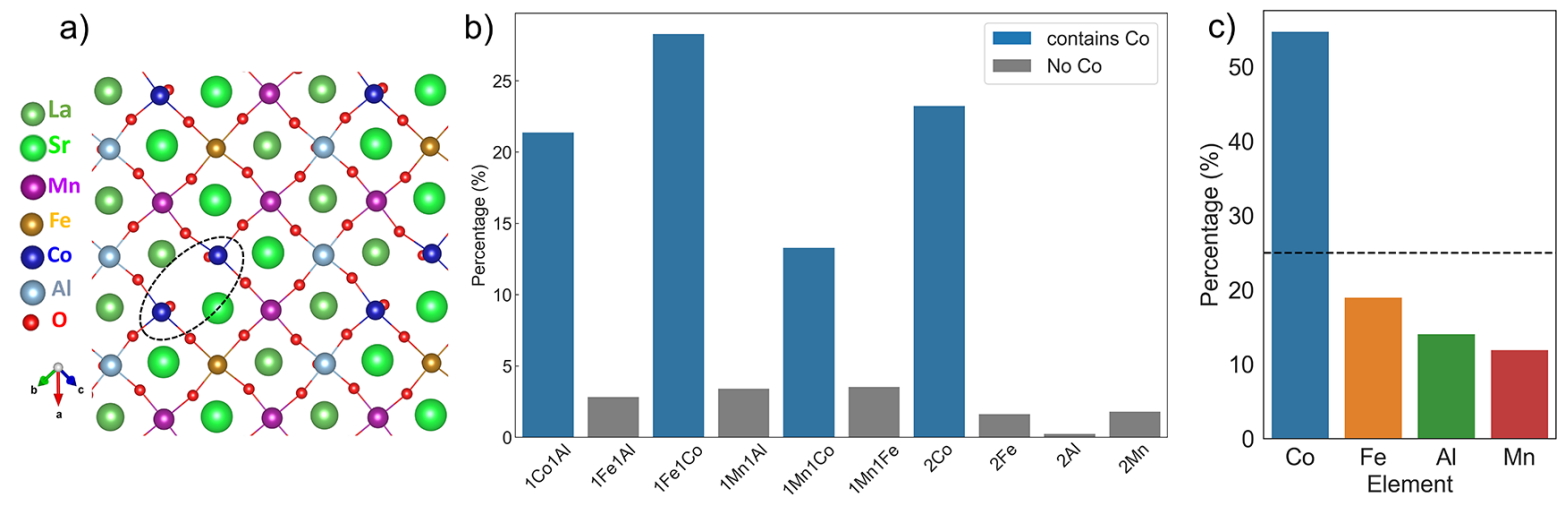
(a) Local ordering in vacancy structures is defined as *V*_O_ FNN {B} site element combinations and one example of Co–Co in the [011] plane is circled. (b) *V*_O_ FNN {B} combination statistics for accepted stable-stage vacancy structures; the Co-containing combinations are plotted in blue while others are in gray. (c) Elemental occurrences for all four {B} site elements, where dotted lines represent 25% random limits.

As for vacancy structures, we are more interested in the elemental preference around the vacancy site; thus, we define *V*_O_ FNN {B} site element combinations as local ordering for the vacancy-present system. An example of a Co–Co combination is circled in Figure 6a in the [011] view. In contrast to bulk cases, the combination statistics of accepted stable-stage vacancy structures shown in Figure 6b indicate predominant Co-containing combinations around the *V*_O_ site, which leads to over 50% Co elemental occurrences well above the random limit of 25%, as shown in Figure 6c. However, this should not be taken as a conflict with the entropy-stabilized single-phase argument of compositionally complex materials, and the result here merely suggests a phenomenological description of vacancy formation: the vacancy site will most probably form at a position where its neighbors contain at least one Co ion. Upon vacancy formation, by referring to the combinations listed in the figure, most of the *V*_O_ FNN sites contain only one redox-active Co, which means that long-range electron transfer is a common phenomenon in LS_MFCA. In contrast to the case reported for SrFeO_3−δ_,^50^ the electrons are transferred to the second or further nearest {B}-site neighbor Co, not due to d-orbital splitting but due to element distributions from cation mixing. From the discussions above, DFT-MC has proven to be capable of capturing both statistically random structures and vacancy formation-related local ordering in CCPOs.

### 3.3 Redox-Active Element and Possible Mechanisms

The neutral *V*_O_ formation leaves two electrons in the host lattice. Determining the redox-active element is critical for understanding the mechanism of oxygen loss and regain in the STCH generation process. In a simple ABO_3_ lattice, which B element will receive these electrons and become redox active is usually straightforward. However, for a system with four elements mixed at {B} sites and two elements mixed at {A} sites, the question becomes complicated. One may simply refer to the conclusion from *E*_v_^f^ of single-component perovskites, as shown in Figure S4, that if Fe^4+^ (HS, 3d^4^: t_2g_^3^e_g_^1^), Mn^4+^ (HS, 3d^3^: t_2g_^3^e_g_^0^), or Co^4+^ (IS, 3d^5^: t_2g_^4^e_g_^1^) exist, these elements will probably be redox-active due to their lower *E*_v_^f^. Though this is a reasonable and empirical attempt, computational and experimental results are counterintuitive.

**Figure 7:**
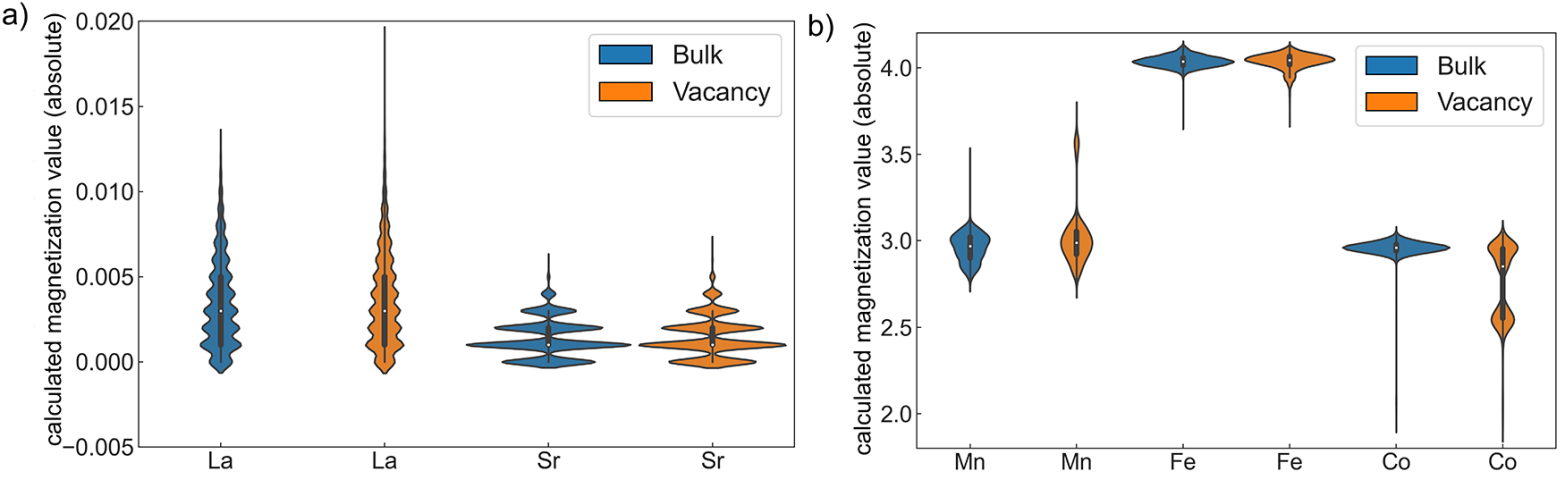
Calculated absolute magnetization value distributions for cations in LS_MFCA are plotted in a violin plot for both bulk (blue) and vacancy (orange) structures. (a) The {A} site element shows no significant magnetization value change between bulk and vacancy structures. (b) {B} site elements in the vacancy structures show that around half of the Co atoms have experienced a magnetization value downshift. Co is the dominant redox-active element, despite some outliers showing the change of Mn or Fe magnetization values.

Oxidation state differences in the bulk and vacancy structures can be directly reflected by DFT-calculated magnetization values (magnetic moment in μ_B_), as shown in Figure 7. The absolute magnetization values per element for the bulk configurations are shown in blue and those for the vacancy configurations are shown in orange. While no {A} site elements show obvious magnetization value changes (<0.01), around half of the Co atoms in the {B} site show a downshift of their values despite some outliers. To determine the charge state in LS_MFCA, the correlations between the GGA + *U* computed magnetic moment of B-site elements in single-component ABO_3_ (A = La, Sr; B = Mn, Fe, Co) and their oxidation states were used as references (Table S1). With the choice of *U*_eff_ = 3, the calculated magnetization values of 3.5/3.0 μ_B_ for Mn are consistent with the loss of unpaired electrons from Mn^3+^(t_2g_^3^e_g_^1^) to Mn^4+^ (t_2g_^3^e_g_^0^) and 4.0/3.6μ_B_ for Fe is consistent with its loss of unpaired electrons from Fe^3+^(t_2g_^3^e_g_^2^) to Fe^4+^(t_2g_^3^e_g_^1^). These agree well with previously reported results.^50,53,54^ The magnetization values of Co ions can be ranked from low-spin (LS) Co^3+^(t_2g_^6^e_g_^0^), high-spin (HS) Co^2+^(t_2g_^5^e_g_^2^), intermediate-spin (IS) Co^4+^, and HS Co^3+^(t_2g_^4^e_g_^2^) with calculated magnetic moment of 0.0/2.5/2.6/3.0μ_B_, in accordance with other theoretical works.^42,55^ As shown in Figure 7, 3.0μ_B_ for Mn^4+^ and 4.0μ_B_ for Fe^3+^ did not show any changes in LS_MFCA upon oxygen vacancy formation. Only 3.0μ_B_ for Co^3+^ split into two values, namely 3.0μ_B_ and ∼2.5μ_B_, indicating that some Co elements experienced a Co^3+^ (HS, 3d^6^: t_2g_^4^e_g_^2^) to Co^2+^ (HS, 3d^7^: t_2g_^5^e_g_^2^) change, showing redox activity. The partial density of states (DOS) of the 3d orbitals is plotted for a randomly selected stable-stage bulk SG 167 structure in Figure S5. This shows that the Co element has the most pronounced low-energy unfilled conduction band and is possibly the most redox-active element during thermal reduction for this selected structure. This is also confirmed by both in situ X-ray photoelectron spectroscopy (XPS) and ex situ scanning transmission electron microscopy (STEM)-electron energy loss spectroscopy (EELS) in calibration with standard compounds that Mn and Fe remained 4+ and 3+, respectively, and only Co is redox-active in the presence of Co^2+^ during thermal reduction.^21^

**Figure 8:**
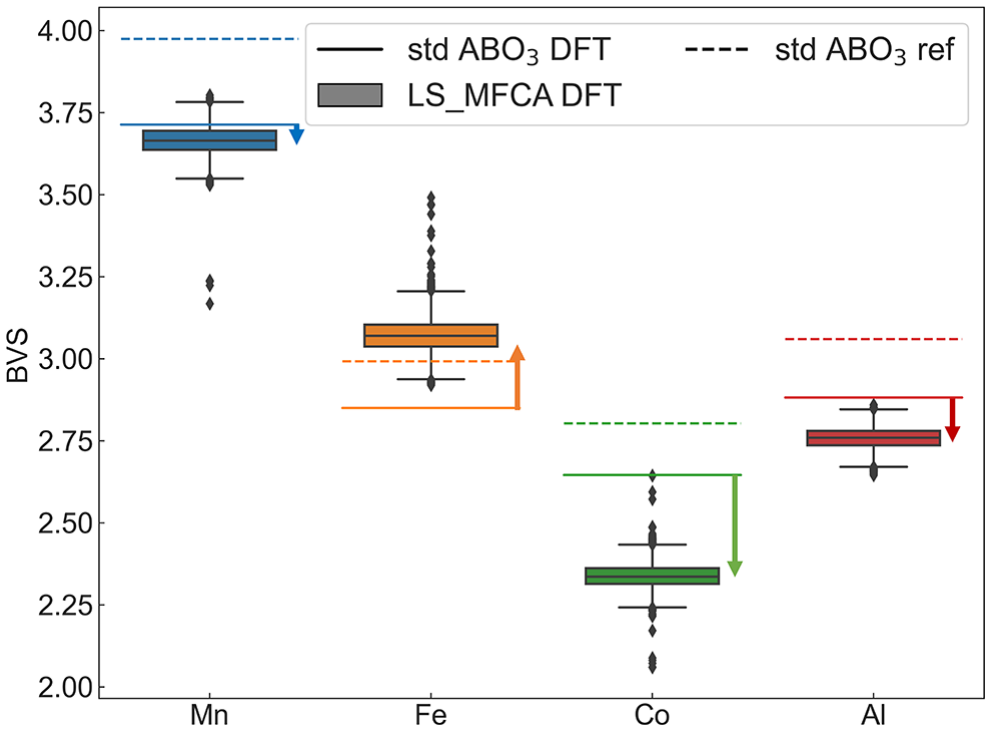
Calculated BVS values are shown in a boxplot for all Mn-, Fe-, Co-, and Al-centered octahedrons in the saved LS_MFCA bulk structures. The dashed lines are the BVS determined from bond lengths reported by experimental measurements (SrMnO_3_,^32^ LaFeO_3_,^63^ LaCoO_3,_^64^ and LaAlO_3_^65^). The solid lines represent the BVS values based on the bond lengths obtained from DFT calculations. Co shows the largest deviation.

The above analysis overturns the empirical argument from single-component vacancy formation energy. In addition to the electronic origin of the preference for Co^3+^ redox activity over Mn^4+^, there is another structural origin that accompanies multication mixing. A possible reason for the preference of Co in vacancy configurations is the Co–O bond stretching effect. Neutral vacancy formation is confirmed to cause volume expansion in many oxides, and a tensile stress in the bulk will lower the vacancy formation energy.^56−58^ For CCPOs, local bond stretching may create extra space to favor *V*_O_ formation. In order to capture the local bond distortion and strength changes, the bond valence sum (BVS) descriptor developed by Brown et al.^59−61^ was adopted:
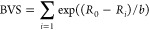
6where BVS is the bond valence sum in valence units (v.u.), *R*_*i*_ is the bond length of the {B}–O bond, *R*_0_ is an arbitrary bond length fitted for each pair of atoms, *b* is a fitted parameter, and is the summation over all six bonds of B-centered octahedron. The BVS for each cation in its single-component structure is usually close to its oxidation number and a lower value is considered to be due to an elongated bond with weakened bond strength and vice versa. For example, using the tabulated parameters listed in Table S2 (developed by fitting a large number of experimental structure data^62^), the BVS using experimentally reported bond lengths for Mn^4+^ in SrMnO_3_, Fe^3+^ in LaFeO_3_, Co^3+^ in LaCoO_3_, and Al^3+^ in LaAlO_3_ are close to 4.0, 3.0, 2.8, and 3.0 (dashed lines in Figure 8), respectively. These values are plotted as dashed lines serving as a general reference to show the appropriate choice of parameters.^32,63−65^ For their DFT-computed relaxed structures, which will serve as computational references for LS_MFCA (solid lines in Figure 8), the bond lengths are consistently longer due to the GGA underbinding crystal. The distributions of BVS in the LS_MFCA system are plotted in a boxplot in the same figure, where the Co BVS distribution is significantly lower than that of the LaCoO_3_ DFT reference, indicating distortion-induced bond elongation and weakening. Meanwhile, the BVS for Mn and Al in LS_MFCA is slightly lower than the DFT reference, while the BVS for Fe is increased. This observation corresponds to slightly weakened Mn–O and Al–O bonds and strengthened Fe–O bonds. Hence, we claim that the Co preference in the vacancy configurations is due to the Co–O bond stretching effect.

Based on the above analysis, the 3:1 ratio of La^3+^ and Sr^2+^ in the {A} site provides the background for the 4+ {B} site element to arise through d-orbital electron competition. By mixing the {B} site Mn, Fe, Co, and Al, Mn is the element that takes the 4+ oxidation state to compensate for the charge neutral requirement, Fe element takes all of the compression at the {B} site inside the lattice, Co is the redox-active element and holds the most elongation and Al provides extra mixing entropy that stabilizes the system.

### 3.4 Enthalpy and Entropy of Reduction

Two important oxygen vacancy formation properties, the enthalpy and entropy of reduction, determine the thermodynamics driving force of the STCH material.^66^ They can be obtained based on the measurements of the oxygen δ over a range of temperatures at different *P*_O_2__ conditions, assuming that the equilibrium conditions of eq 1 have been reached.^67,68^ According to the van’t Hoff method,^67,69,70^ the enthalpy and entropy of reduction, at a given temperature and *P*_O_2__ can be expressed as
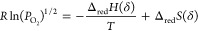
7where *R* is the gas constant, and Δ_red_*H*(δ) and Δ_red_*S*(δ) are assumed to be independent of *T*.

To facilitate a direct comparison with the experiments, we obtained these thermodynamic properties from our calculations. For single-value *E*_v_^f^ ABO_3_ with linear vacancy interaction behavior (low-entropy interacting model, denoted as LE-Int), an analytical solution can be obtained through the free-energy change expression Δ*G*_v_^f^(*T*,*P*_O_2__,δ) = *E*_v_^f^ + αδ + Δμ_O_(*T*,*P*_O_2__) and the nonstoichiometry relation δ/(3−δ) = exp(−Δ*G*_v_^f^/*k*_B_*T*). By applying the simplified assumption of an ideal constant *C*_p_, the enthalpy and entropy of reduction can be obtained as (derivation details in Supporting Information (SI), Section S1)
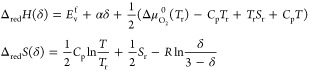
8Δ_red_*H*(δ) has a temperature-dependent term coming from , with a major contribution from *E*_v_^f^ + αδ that almost changes linearly with an increase of δ. Δ_red_*S*(δ) has a clear  term and trend with a temperature dependence of .

**Figure 9:**
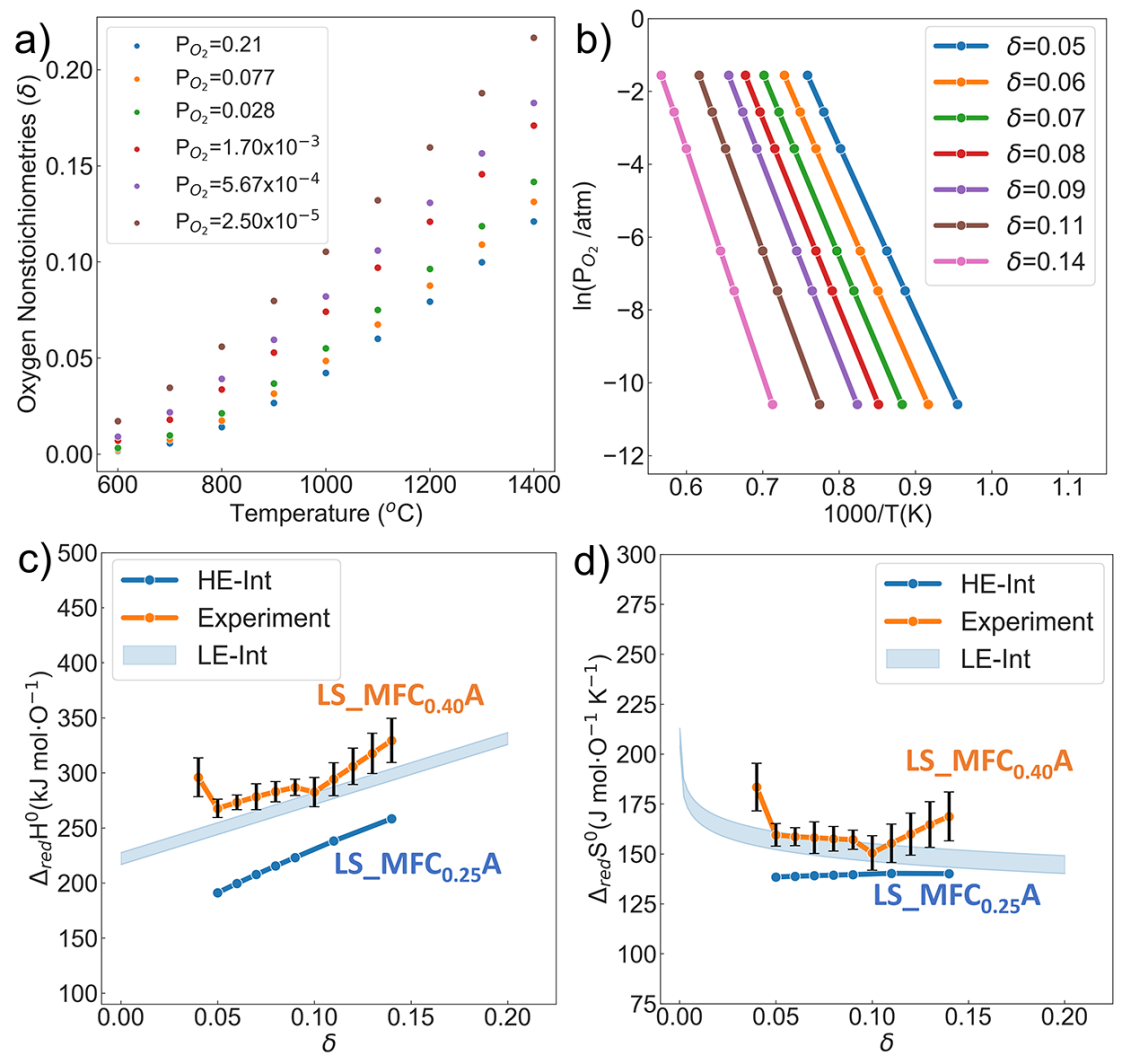
(a) Calculated oxygen nonstoichiometry of LS_MFCA with respect to temperature at given *P*_O_2__ conditions. (b) The van’t Hoff method of an Arrhenius plot of ln(*P*_O_2__) with respect to 1000/*T* at a specified δ value for extracting the enthalpy and entropy of reduction. (c) Calculated standard enthalpy and (d) entropy of reduction for LS_MFCA marked as HE-Int; the experimentally measured values and error bars are reported by Zhang et al. for (La_0.8_Sr_0.2_)(Mn_0.2_Fe_0.2_Co_0.4_Al_0.2_)O_3_.^21^ The blue shaded area is from the analytical model in eq 8, noted as the LE-Int result, with *E*_v_^f^ = 2.12 eV, whose lower and upper bounds correspond to *T*_r_ = 800 °C and *T*_r_ = 1350 °C.

For a distributed *E*_v_^f^, such a simple derivation cannot be obtained. Therefore, we created a “digital twin” of the experiment. The statistical model is explained in the Method section and the result is marked as “HE-Int” in Figure 9. We first applied it to predict the nonstoichiometry δ over a range of temperature *T* and oxygen partial pressure *P*_O_2__, as shown in Figure 9a. After fitting the δ−*T* data with a polynomial, an Arrhenius plot of ln(*P*_O_2__) with respect to 1000/*T* at the selected δ value is plotted with linear fitting in Figure 9b, where the Δ_red_*H*(δ) and Δ_red_*S*(δ) values can be extracted from the slope and *y*-axis intersection. They are plotted on a per mole oxygen basis in Figure 9c,d, and the experimentally measured values for (La_0.8_Sr_0.2_)(Mn_0.2_Fe_0.2_Co_0.4_Al_0.2_)O_3_ with error bars are included for direct comparison.

The blue shaded area shows the results from the analytical model in eq 8, with *E*_v_^f^ = *E̅*_vac_ – *E̅*_bulk_ + μ_O,FERE_ = 2.12 eV, as the average value of *E*_v,CCPO_^f^ distribution and lower and upper bounds correspond to *T*_r_ = 800 °C and *T*_r_ = 1350 °C. The analytical model captures the trend and values of Δ_red_*H*(δ) and Δ_red_*S*(δ) with relatively good agreement at a small δ region, which deviates from the experimental values when δ of LS_MFCA exceeds 0.1. Meanwhile, the overall increasing trend in Δ_red_*H*(δ) and steady behavior in Δ_red_*S*(δ) are properly captured with the HE-Int model, and the calculated values are systematically smaller than the measurements, especially for Δ_red_*H*(δ), which is around 50 kJ/mol lower than the analytical result.

Despite the compositional difference in Co content between the experiment and model that may lead to the systematic difference, the main discrepancy in Δ_red_*H*(δ) can be mainly ascribed to the overestimation of δ when considering the inclusion of distribution: the HE-Int model overestimates δ in lower temperature regions and results in a narrower temperature range (thus a broader 1/T range) at each given δ, leading to a flatter slope in the Arrhenius plot with respect to 1000/*T*, and thus a lower Δ_red_*H*(δ) value. Improving the model by considering the distribution shape change upon *V*_O_ formation, i.e., jumping out of the independent *V*_O_ occurrence assumption as mentioned in Section 3.1, is a feasible attempt. The lower Δ_red_*S*(δ) can be relieved by including more possible contributions such as vibrational, orbital, spin, and charge entropy in the model, but the computational cost will increase dramatically. The δ ≈ 0.045 anomalies in the experimental curve that are believed to occur at transitions in electronic behavior cannot be reproduced for these factors and are not included in the original model.^67^ Since the HE-Int underestimation in Δ_red_*H*(δ) and Δ_red_*S*(δ) are systematic and originates mainly from the treatment of *E*_v_^f^ distribution therein, with the correct trend it captures, relative STCH performance comparison between different compositions from a computational perspective is still possible.

## 4 Conclusions

In summary, by examining the example CCPO material LS_MFCA, the DFT + *U* calculations combined with the MC method in this work are proven to be able to reveal not only the redox-active element and oxidation state of each {B} site cation through magnetization value comparison but also the *V*_O_ FNN {B} site element preference, which is not usually viable through nonstatistical methods. With a system containing both Co^3+^ and Mn^4+^, which is different from empirical expectations based on single-component vacancy formation energy, Co is redox-active and the *V*_O_-preferred FNN elements. The origin of this discrepancy can be explained by the fact that Co–O bonds are much more stretched (weakened) than Mn–O and Al–O bonds, while Fe–O bonds are compressed (strengthened). The local excess space due to Co–O bond stretching outpaced the tendency to generate vacancies near Mn^4+^. The bond length deviation is captured by the BVS descriptor, which shows discrepancies from its ideal values due to distortions. Because of the structural complexity and ionic radius differences between the {A} and {B} sites, extra attention is required to understand the role of each element compared to HEAs and HEOs. The disorder-enabled bond fluctuation reported in this study using the DFT-MC method can also be extrapolated to other CCPO systems.

Moreover, based on the DFT-MC energy evolution path and accepted stable-stage structures, the definition of vacancy formation energy for a mixed-cation system is proposed. With this distribution-based formation energy and redox-active element, the enthalpy and entropy of reduction can be extracted and compared with experimental measurements. Even though the absolute value of the enthalpy of reduction is underestimated, the value for the entropy of reduction and both their trends are comparable. A comparison between different compositions by using the HE-Int model still provides relative STCH performance differences computationally, which could benefit the material selection. The DFT-MC method provides an enclosed routine of vacancy formation energy calculations, redox and mechanism studies, and thermodynamical property prediction, which we hope could serve as prototypical high-throughput benchmarks for compositionally complex materials. Such a practice is critical in material searching and composition prediction for applications that favor specific ranges of enthalpy and entropy, i.e., studies have shown that intermediate enthalpy and large entropy work better for the STCH process.^67,71^ Though the DFT-MC method is still relatively slow at the current stage, an alternative method of using machine-learned interaction potentials (MLIPs) could possibly take the job and speed up the sampling process.^72^ We envision that computational screening of compositionally complex materials can wield its power to predict functional properties and provide guidance for what to synthesize in the future.

## Supporting Information Available

Additional details and information on the ABO_3_ case and van’t Hoff relation derivation; α determination and other DFT setup (setup1) parameter choices and resulting δ prediction; adopted perovskite structures and example processing results; oxygen-refilled structure energy and resulting vacancy formation energy distributions; single-component vacancy formation energies for ABO_3_; calculated magnetization values and oxidation states, and BVS tabulated parameters (PDF)
