# Supplementary material for: Local Ordering, Distortion, and Redox Activity in (La0.75Sr0.25)(Mn0.25Fe0.25Co0.25Al0.25)O3 Investigated by a Computational Workflow for Compositionally Complex Perovskite Oxides
Source: Chem Mater. 2024 May 13;36(10):4990–5001. doi: 10.1021/acs.chemmater.3c03038 (PMC11138530; doi:10.1021/acs.chemmater.3c03038)
Supplement: Supplementary file 1 — cm3c03038_si_001.pdf [file cm3c03038_si_001.pdf]

Supplementary Material

The Local Ordering, Distortion, and Redox Activity in $(La_{0.75}Sr_{0.25})(Mn_{0.25}Fe_{0.25}Co_{0.25}Al_{0.25})O_3$ Investigated by a Computational Workflow for Compositionally Complex Perovskite Oxides

Boyuan Xu[a], Jiyun Park[b], Dawei Zhang[c], Hector A De Santiago[d], Wei Li[d], Xingbo Liu[d], Jian Luo[c,e], Stephan Lany[f,*], Yue Qi[b,*]

[a] Department of Physics, Brown University, Providence, Rhode Island 02912, USA
[b] School of Engineering, Brown University, Providence, Rhode Island 02912, USA
[c] Program in Materials Science and Engineering, University of California San Diego, La Jolla, CA 92093, USA
[d] Department of Mechanical and Aerospace Engineering, Benjamin M. Statler College of Engineering and Mineral Resources, West Virginia University, Morgantown, WV 26506, USA
[e] Department of NanoEngineering, University of California San Diego, La Jolla, CA 92093, USA
[f] Materials Science Center, National Renewable Energy Laboratory, Golden, Colorado 80401, USA

[*]Corresponding authors: Stephan.Lany@nrel.gov; yueqi@brown.edu

Section S1. ABO_3 case van't Hoff relation derivation:

Starting from equations

$$\Delta G_v^f(T,P_{O_2},\delta) = E_v^f + \alpha\delta + \Delta\mu_O(T,P_{O_2}) \quad (1)$$

$$\frac{\delta}{3-\delta} = \exp\left(-\Delta G_v^f/(k_B T)\right) \quad (2)$$

Replace the ΔG_v^f with equation (1):

$$-k_B T \ln\frac{\delta}{3-\delta} = \Delta\mu_O(T,P_{O_2}) + E_v^f + \alpha\delta \quad (3)$$

Expand $\Delta\mu_O(T,P_{O_2})$ into its components:

$$-k_B T \ln\frac{\delta}{3-\delta} = \frac{1}{2}\left(\Delta\mu_{O_2}^0(T_r) + \int_{T_r}^{T} C_p dT - T\int_{T_r}^{T}\frac{C_p}{TdT} - (T-T_r)S_r + k_B T\ln P_{O_2}\right) + E_v^f + \alpha\delta \quad (4)$$

By applying simplified assumption of ideal constant C_p, the equation becomes:

$$-k_B T \ln\frac{\delta}{3-\delta} = \frac{1}{2}\left(\Delta\mu_{O_2}^0(T_r) + C_p(T-T_r) - TC_p\ln\frac{T}{T_r} - (T-T_r)S_r + k_B T\ln P_{O_2}\right) + E_v^f + \alpha\delta \quad (5)$$

Rearrange the equation into T dependent terms and independent terms:

$$-k_B T\ln(P_{O_2})^{\frac{1}{2}} = E_v^f + \alpha\delta + \frac{1}{2}\left(\Delta\mu_{O_2}^0(T_r) - C_p T_r + T_r S_r\right)$$

$$+\frac{1}{2}\left(C_p T - C_p T\ln\frac{T}{T_r} - TS_r + 2k_B T\ln\frac{\delta}{3-\delta}\right) \quad (6)$$

Divide both sides of the equation by $-T$:

$$k_B\ln(P_{O_2})^{\frac{1}{2}} = -\frac{E_v^f + \alpha\delta + \frac{1}{2}\left(\Delta\mu_{O_2}^0(T_r) - C_p T_r + T_r S_r\right)}{T} - \frac{1}{2}\left(C_p - C_p\ln\frac{T}{T_r} - S_r + 2k_B\ln\frac{\delta}{3-\delta}\right) \quad (7)$$

Doing a unit conversion from eV to J/mol and replace k_B with R:

$$R\ln(P_{O_2})^{\frac{1}{2}} = -\frac{E_v^f + \alpha\delta + \frac{1}{2}\left(\Delta\mu_{O_2}^0(T_r) - C_p T_r + T_r S_r\right)}{T} - \frac{1}{2}\left(C_p - C_p\ln\frac{T}{T_r} - S_r + 2R\ln\frac{\delta}{3-\delta}\right) \quad (8)$$

Take derivative about T for both sides:

$$R\frac{d\ln(P_{O_2})^{\frac{1}{2}}}{dT} = -\frac{E_v^f + \alpha\delta + \frac{1}{2}\left(\Delta\mu_{O_2}^0(T_r) - C_p T_r + T_r S_r\right)}{T^2} + \frac{C_p}{2T} \quad (9)$$

The expression for enthalpy of reduction:

$$\Delta_{\text{red}}H(\delta) = E_v^f + \alpha\delta + \frac{1}{2}\left(\Delta\mu_{O_2}^0(T_r) - C_p T_r + T_r S_r + C_p T\right) \quad (10)$$

Then the entropy of reduction becomes:

$$\Delta_{\text{red}}S(\delta) = R\ln(P_{O_2})^{\frac{1}{2}} + \frac{\Delta_{\text{red}}H}{T} = \frac{1}{2}C_p ln\frac{T}{T_r} + \frac{1}{2}S_r - Rln\frac{\delta}{3-\delta} \quad (11)$$

Section S2. α determination and other DFT setup (setup1):

To obtain linear interaction coefficient, similar setup as in DFT-MC sampling is used for supercell of $R\bar{3}c$ $LaCoO_3$, while the electronic and atomic relaxation convergence criteria were selected to be 1×10^{-6} and 0.01 eV Å^{-1}. High spin (HS) with antiferromagnetic (AFM) alignment was adopted for Co in accordance with MC-DFT result of HS Co. The E_v^f was calculated for structures containing one vacancy in supercells with 40 atoms, 80 atoms and 160 atoms, respectively.

Other DFT setup (setup1: U_{Co}=4.0 eV, U_{La}=0 eV, standard O pseudopotential, 500 eV cutoff), as typically adapted for V_O calculations, was tested and shown in Figure S3a and marked in black. Around 0.2 eV difference in E_v^f was captured and a linear fitting of setup1 E_v^f gave an interaction coefficient of $\alpha = 5.20$. The qualitative trends in E_v^f with vacancy concentration do not depend on U selections and can be captured by both DFT-MC and setup1.

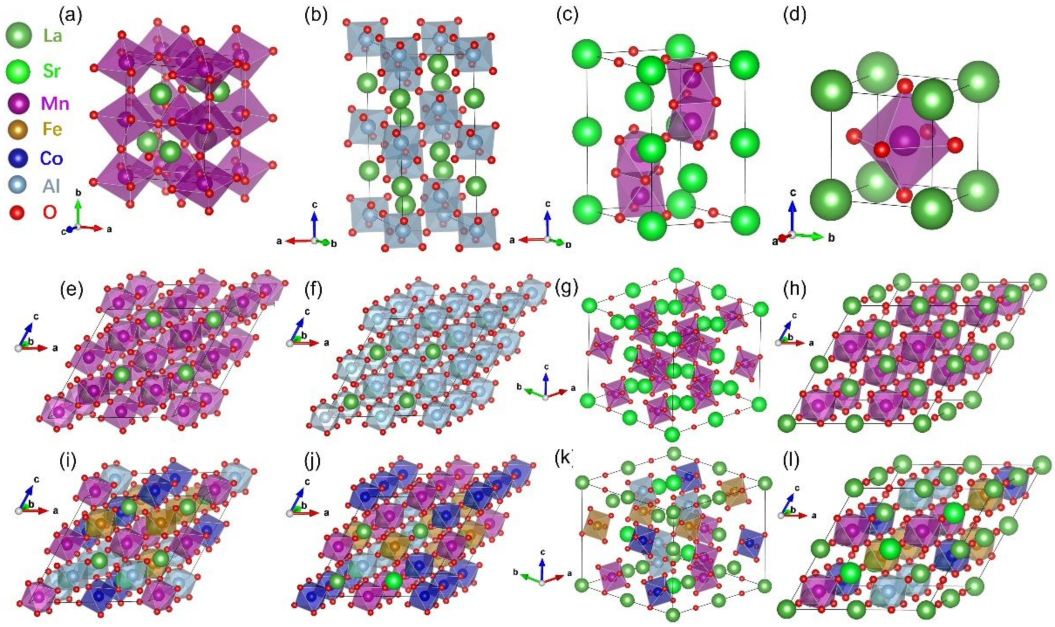

Figure S1 Four most common perovskite symmetries with representative compound, a). $LaMnO_3$ Pnma (SG 62), b). $LaAlO_3$ R$\bar{3}$c (SG 167), c). $SrMnO_3$ P6_3/mmc (SG 194) and d). $LaMnO_3$ Pm$\bar{3}$m (SG 221), were used as initial perovskite structures (the "seed") to generate e,f,g,h). the 80-atom supercell, then all four supercells were transformed to i,j,k,l). the cation-randomly-mixed LS_MFCA supercells. A test run with both atomic and volume relaxation is conducted first for over 50 iterations, the average volume is determined for later official DFT-MC sampling process which only includes atomic relaxation.

Structure Information:

	Original lattice		Simulation Cell	
	Lattice Parameter (Å)	Equivalency in a cubic like lattice	Lattice Parameter (Å)	Equivalency in a cubic like lattice
Pnma	$a = 5.87$<br>$b = 7.78$<br>$c = 5.59$<br>$\alpha = \beta = \gamma = 90°$	$a = [10\bar{1}]$<br>$b = 2[010]$<br>$c = [101]$	$a = c = 11.02$<br>$b = 11.15$<br>$\alpha = \gamma = 59.61°$<br>$\beta = 59.64°$	$a = 2[10\bar{1}]$<br>$b = 2[01\bar{1}]$<br>$c = 2[110]$
R$\bar{3}$c	$a = b = 5.41$<br>$c = 13.19$<br>$\alpha = \beta = 90°$<br>$\gamma = 120°$	$a = [1\bar{1}0]$<br>$b = [011]$<br>$c = 2[\bar{1}\bar{1}1]$	$a = b = c = 11.02$<br>$\alpha = \beta = \gamma = 60.13°$	$a = 2[101]$<br>$b = 2[110]$<br>$c = 2[011]$
P6_3/mmc (Face shared octahedrons)	$a = b = 5.52$<br>$c = 9.25$<br>$\alpha = \beta = 90°$<br>$\gamma = 120°$	$a = [1\bar{1}0]$<br>$b = [01\bar{1}]$<br>$c = 1.37[111]$	$a = b = 11.10$<br>$c = 10.82$<br>$\alpha = \beta = 75.14°$<br>$\gamma = 120°$	$a = 2[1\bar{1}0]$<br>$b = 2[01\bar{1}]$<br>$c = 2[110]$
Pm$\bar{3}$m	$a = b = c = 3.94$<br>$\alpha = \beta = \gamma = 90°$	$a = [100]$<br>$b = [010]$<br>$c = [001]$	$a = b = c = 11.03$<br>$\alpha = \beta = \gamma = 60°$	$a = 2[10\bar{1}]$<br>$b = 2[01\bar{1}]$<br>$c = 2[110]$

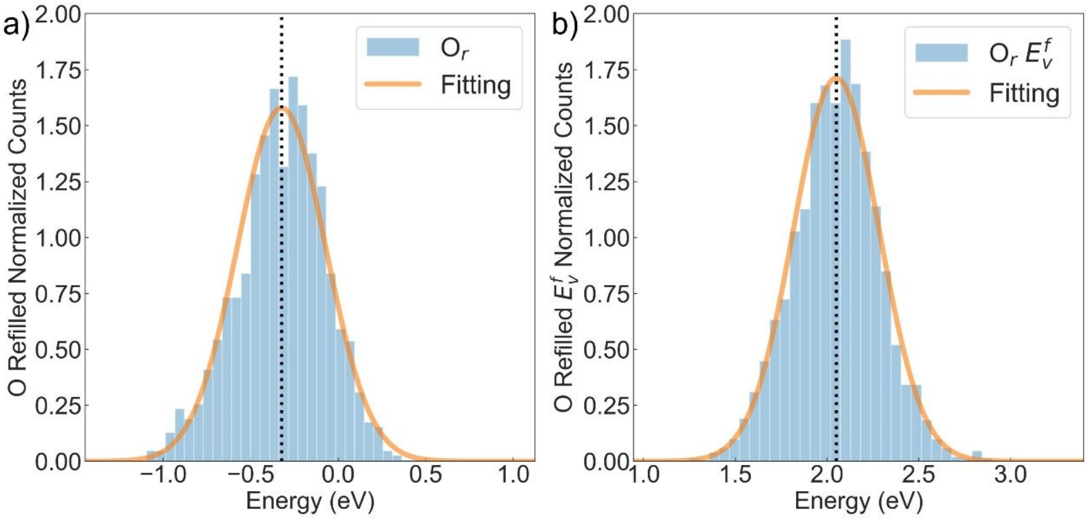

Figure S2 Fill vacancy structures with oxygen atom and obtain vacancy formation energy. a) The energy of refilled structures is fitted with a gaussian function that has energy average at -594.48 eV with a standard deviation of 0.25 eV. b) Similar operation is taken for $E_{v,r}^{f}$ distribution that results in an energy average at 2.05 eV with standard deviation of 0.24 eV.

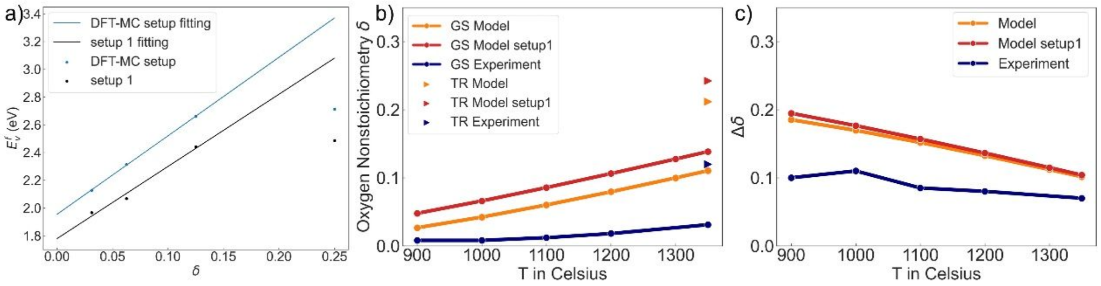

Figure S3 a). The vacancy interaction coefficient α is obtained from linear fitting of varying size $LaCoO_3$ vacancy formation energy. Only E_v^f of 40-atom, 80-atom and 160-atom supercell are included in the fitting. The fitted slope for DFT-MC setup gives $\alpha = 5.66$ with an intercept at 1.95 eV, while the fitted slope for setup 1 (U_{Co}=4.0 eV, U_{La}=0 eV, standard O pseudopotential, 500 eV cutoff) gives $\alpha = 5.20$ with an intercept at 1.78 eV. b). Setup 1 gives a higher predicted δ trend as shown in red, which doesn't relieve the discrepancy between theoretical prediction and experimental measurements. c). $\Delta\delta$ is not significantly affected by switching from DFT-MC setup to setup1.

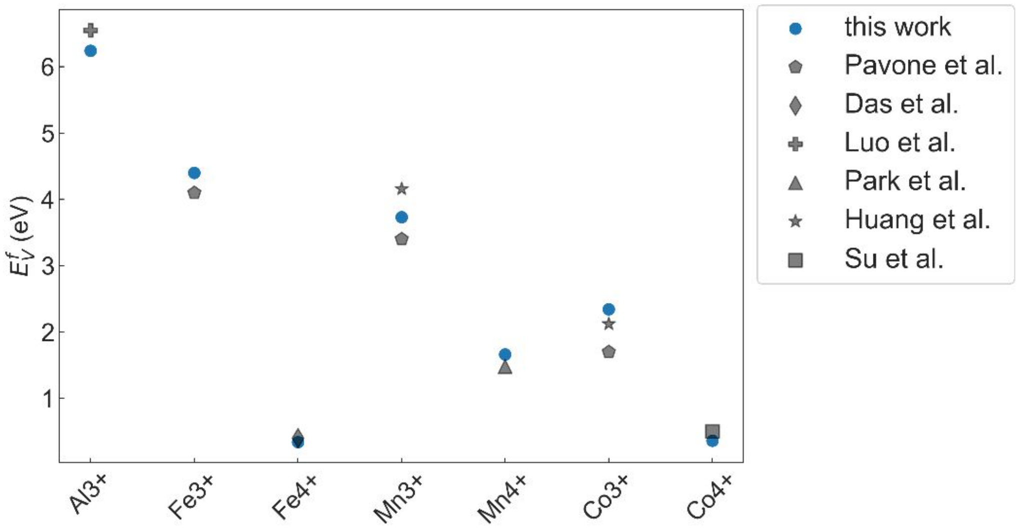

Figure S4 Single component vacancy formation energies for ABO_3 (A=La, Sr; B=Mn, Fe, Co, Al) using GGA+U method and compared with published works.[1,2,3,4,5,6]

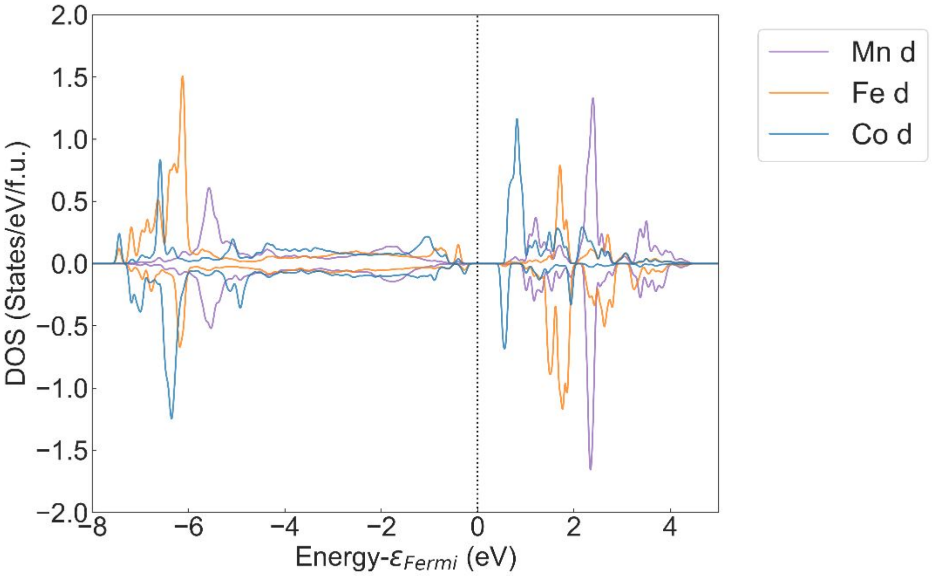

Figure S5 The PDOS per formula unit was plotted for d-orbital of Mn, Fe and Co with x=0 set to be the fermi energy for a randomly selected saved stable stage bulk SG 167 structure.

{B} site ion	Ion types	Electron Configuration	Magnetization values μ_B (absolute)	Reference values μ_B (absolute)
Mn ion	Mn^{3+}	$t_{2g}^3 e_g^1$	3.5	3.43[7]
	Mn^{4+}	$t_{2g}^3 e_g^0$	3	2.8[8]
Fe ion	Fe^{3+}	$t_{2g}^3 e_g^2$	4	4.23[2]
	Fe^{4+}	$t_{2g}^3 e_g^1$	3.6	3.61[2]
Co ion	Co^{2+} (High Spin)	$t_{2g}^5 e_g^2$	2.5	2.5[9]
	Co^{3+} (High Spin)	$t_{2g}^4 e_g^2$	3	2.98[9]
	Co^{3+} (Low Spin)	$t_{2g}^6 e_g^0$	0	0[9]
	Co^{4+} (Intermediate Spin)	$t_{2g}^4 e_g^1$ or $t_{2g}^5 e_g^0$	2.6	2.6[10]

Table S1 Calculated magnetization value and oxidation state relation from ABO_3 (A=La, Sr; B= Mn, Fe, Co) GGA+U calculations.

**Cation**	**Anion**	$\boldsymbol{R_0}$	***b***	**Compound**	**BVS (Exp.)**	**BVS (DFT)**
Mn^{4+}	O^{2-}	1.750	0.374	$SrMnO_3$	3.975	3.715
Fe^{3+}	O^{2-}	1.766	0.360	$LaFeO_3$	3.064	2.850
Co^{3+}	O^{2-}	1.655	0.364	$LaCoO_3$	2.819	2.661
Al^{3+}	O^{2-}	1.634	0.390	$LaAlO_3$	3.033	2.882

Table S2 Tabulated values for R_0 and b from Gagne *et al.*.[11] Experiment BVS are from the ICSD reported high-quality structures ($SrMnO_3$,[12] $LaFeO_3$,[13] $LaCoO_3$[14] and $LaAlO_3$[15]). The DFT BVS values are from GGA+U result. Low spin $LaCoO_3$ is selected as the ground state for Co^{3+}.

Supplementary References:

(1) Pavone, M.; Ritzmann, A. M.; Carter, E. A. Quantum-mechanics-based design principles for solid oxide fuel cell cathode materials. *Energy & Environmental Science* **2011**, *4* (12), 4933-4937. DOI: 10.1039/C1EE02377B.

(2) Das, T.; Nicholas, J. D.; Qi, Y. Long-range charge transfer and oxygen vacancy interactions in strontium ferrite. *Journal of Materials Chemistry A* **2017**, *5* (9), 4493-4506. DOI: 10.1039/C6TA10357J.

(3) Luo, X.; Wang, B.; Zheng, Y. First-principles study on energetics of intrinsic point defects in LaAlO3. *Physical Review B* **2009**, *80* (10), 104115. DOI: 10.1103/PhysRevB.80.104115.

(4) Park, J.; Wu, Y.-N.; Saidi, W. A.; Chorpening, B.; Duan, Y. First-principles exploration of oxygen vacancy impact on electronic and optical properties of ABO3−δ (A = La, Sr; B = Cr, Mn) perovskites. *Physical Chemistry Chemical Physics* **2020**, *22* (46), 27163-27172. DOI: 10.1039/D0CP05445C.

(5) Huang, W. L.; Zhu, Q.; Ge, W.; Li, H. Oxygen-vacancy formation in LaMO3 (M=Ti, V, Cr, Mn, Fe, Co, Ni) calculated at both GGA and GGA+U levels. *Computational Materials Science* **2011**, *50* (5), 1800-1805. DOI: https://doi.org/10.1016/j.commatsci.2011.01.018.

(6) Su, H.-Y.; Sun, K. DFT study of the stability of oxygen vacancy in cubic ABO3 perovskites. *Journal of Materials Science* **2015**, *50* (4), 1701-1709. DOI: 10.1007/s10853-014-8731-0.

(7) Pishahang, M.; Mohn, C. E.; Stølen, S.; Bakken, E. DFT-study of the energetics of perovskite-type oxides LaMO3 (M = Sc–Cu). *RSC Advances* **2012**, *2* (28), 10667-10672. DOI: 10.1039/C2RA21139D.

(8) Edström, A.; Ederer, C. First-principles-based strain and temperature-dependent ferroic phase diagram of SrMnO3. *Physical Review Materials* **2018**, *2* (10), 104409. DOI: 10.1103/PhysRevMaterials.2.104409.

(9) Ritzmann, A. M.; Pavone, M.; Muñoz-García, A. B.; Keith, J. A.; Carter, E. A. Ab initio DFT+U analysis of oxygen transport in LaCoO3: the effect of Co3+ magnetic states. *Journal of Materials Chemistry A* **2014**, *2* (21), 8060-8074. DOI: 10.1039/C4TA00801D.

(10) Lee, J. H.; Rabe, K. M. Coupled Magnetic-Ferroelectric Metal-Insulator Transition in Epitaxially Strained SrCoO3 from First Principles. *Physical Review Letters* **2011**, *107* (6), 067601. DOI: 10.1103/PhysRevLett.107.067601.

(11) Gagné, O. C.; Hawthorne, F. C. Comprehensive derivation of bond-valence parameters for ion pairs involving oxygen. *Acta Crystallogr B Struct Sci Cryst Eng Mater* **2015**, *B71*, 562-578. DOI: 10.1107/s2052520615016297.

(12) Hona, R. K.; Ramezanipour, F. Effect of the Oxygen Vacancies and Structural Order on the Oxygen Evolution Activity: A Case Study of SrMnO3−δ Featuring Four Different Structure Types. *Inorganic Chemistry* **2020**, *59* (7), 4685-4692. DOI: 10.1021/acs.inorgchem.9b03774.

(13) Ivanov, S. A.; Tellgren, R.; Porcher, F.; Ericsson, T.; Mosunov, A.; Beran, P.; Korchagina, S. K.; Kumar, P. A.; Mathieu, R.; Nordblad, P. Preparation, structural, dielectric and magnetic properties of LaFeO3–PbTiO3 solid solutions. *Materials Research Bulletin* **2012**, *47* (11), 3253-3268. DOI: https://doi.org/10.1016/j.materresbull.2012.08.003.

(14) Taguchi, H.; Matsuoka, S.; Kato, M.; Hirota, K. Crystal structure and methane oxidation on perovskite-type (La1-xNdx)CoO3 synthesized using citric acid. *Journal of Materials Science* **2009**, *44* (21), 5732-5736. DOI: 10.1007/s10853-009-3802-3.

(15) Lehnert, H.; Boysen, H.; Schneider, J.; Frey, F.; Hohlwein, D.; Radaelli, P.; Ehrenberg, H. A powder diffraction study of the phase transition in LaAlO3. *Zeitschrift für Kristallographie - Crystalline Materials* **2000**, *215* (9), 536-541. DOI: doi:10.1524/zkri.2000.215.9.536.
